# Correlation Coefficients Between Different Methods of Expressing Bacterial Quantification Using Real Time PCR

**DOI:** 10.3390/ijms13022119

**Published:** 2012-02-16

**Authors:** Bahman Navidshad, Juan Boo Liang, Mohammad Faseleh Jahromi

**Affiliations:** 1 Department of Animal Science, University of Mohaghegh Ardabili, 56199-11367, Ardabil, Iran; E-Mail: bnavidshad@uma.ac.ir; 2 Institute of Tropical Agriculture, University Putra Malaysia, 43400 UPM Serdang, Malaysia; 3 Laboratory of Industrial Biotechnology, Institute of Bioscience, University Putra Malaysia, 43400 UPM Serdang, Malaysia; E-Mail: mohammadfj2006@yahoo.com

**Keywords:** real time PCR, bacterial quantification, absolute and ΔΔCt, Pfaffl equation

## Abstract

The applications of conventional culture-dependent assays to quantify bacteria populations are limited by their dependence on the inconsistent success of the different culture-steps involved. In addition, some bacteria can be pathogenic or a source of endotoxins and pose a health risk to the researchers. Bacterial quantification based on the real-time PCR method can overcome the above-mentioned problems. However, the quantification of bacteria using this approach is commonly expressed as absolute quantities even though the composition of samples (like those of digesta) can vary widely; thus, the final results may be affected if the samples are not properly homogenized, especially when multiple samples are to be pooled together before DNA extraction. The objective of this study was to determine the correlation coefficients between four different methods of expressing the output data of real-time PCR-based bacterial quantification. The four methods were: (i) the common absolute method expressed as the cell number of specific bacteria per gram of digesta; (ii) the Livak and Schmittgen, ΔΔCt method; (iii) the Pfaffl equation; and (iv) a simple relative method based on the ratio of cell number of specific bacteria to the total bacterial cells. Because of the effect on total bacteria population in the results obtained using ΔCt-based methods (ΔΔCt and Pfaffl), these methods lack the acceptable consistency to be used as valid and reliable methods in real-time PCR-based bacterial quantification studies. On the other hand, because of the variable compositions of digesta samples, a simple ratio of cell number of specific bacteria to the corresponding total bacterial cells of the same sample can be a more accurate method to quantify the population.

## 1. Introduction

The gastrointestinal tract (GIT) of chicken contains an array of bacterial populations that are essential for the growth and health of the host animal by influencing intestinal morphology, digestion and absorption processes as well as protecting it against infection [1,2]. However, information on the ecology of the GIT in chicken is rather inadequate. *Lactobacilli* are widespread in the crop and ileum, while obligate anaerobes are the majority in the cecum [3–6]. The gut microbiota is a dynamic population, as it continues to change over time and is affected by the age of the bird, feed and the dietary inclusion of antibiotics and probiotics [7–13].

Bacterial quantification is an important aspect in microbiological studies. Most previous studies in broiler chickens used culture-dependent approaches and have shown that the culturable bacteria in the GIT mostly belong to the *Lactobacilli*, *Enterococci*, and *Enterobacteria*, while *Lactobacilli*, *Enterococci*, *Bacteroides*, and *Clostridia* are the predominant bacteria of the cecum [14–17]. Applications of the traditional culture-dependent methods are limited by their dependence on the inconsistent success of the culture procedures, and some bacteria can be pathogenic or function as a source of endotoxins, causing health problems. Thus, the development of alternative bacterial quantification methods would be useful.

Bacterial quantification based on real-time PCR has helped to overcome the above-mentioned problems. Real-time PCR has been developed for monitoring the amplification reaction. SYBR Green I is a double-stranded DNA binding dye that allows the detection of PCR products, including the DNA extracted from bacterial samples [18,19].

Many of the PCR-based methods for identifying bacteria use agarose gel electrophoresis for end-point recognition. The size of the bands and their intensity can be photographed and integrated, and standard curves are obtained with the use of the NIH image software program. The major disadvantages of agarose gels is the additional time required and the fact that standard curves encompass no more than two log cycles compared to 6 log cycles with real-time PCR.

The bacterial quantification data using real-time PCR is commonly expressed as absolute quantities in units such as copies/g, colony*-*forming unit (CFU)/mL or Log CFU/g of samples such as digesta. The digesta composition, particularly the dry matter content, can vary substantially depending on factors such as feed composition [20–22], dietary particle size [23] or water consumption [24], and without proper homogenisation of the samples, especially when multiple sample are to be pooled before DNA extraction, the final results may be affected. In a pre-experiment, we examined the data on DNA extraction from digesta samples based on differing moisture contents and found that bacterial loads of digesta samples are mainly in the liquid phase of the digesta and that the best absolute unit was expressed as bacterial CFU per mL of digesta liquid content rather than on a dry matter basis [25]. There are also few reports of the loads in individual bacterial groups as a fraction of the total bacterial population [26,27].

Feng *et al.* [28] used the Livak and Schmittgen ΔΔCt method [29] to calculate the relative quantity of different bacterial population relative to the total bacteria mass as a reference (like the role of housekeeping genes in gene expression studies). However, this methodology was originally developed to calculate relative gene expression in reverse transcription-real-time PCR methods, and there are some obvious differences between bacterial population quantification and gene expression studies. For example, it is assumed that the expression of housekeeping genes in tissues is not affected by treatments. However, whether the same is true for total bacterial contents of digesta samples is not known. Thus, it is difficult to determine whether alterations in the microbial populations as reported by Feng *et al.* [28] were due to the experimental treatments or the results of the method of expression of the data.

The objective of the present study was to determine the correlation coefficients between three different methods of expressing the output data for real-time PCR-based bacterial quantification. The four methods examined were: (i) that common absolute method expressed as the cell number of a specific bacteria per mL of liquid phase of digesta; (ii) the Livak and Schmittgen ΔΔCt method; (iii) the Pfaffl equation as a ΔCt-based relative quantification method; and (iv) a simple relative method, expressed as cell number of a specific bacteria as a ratio of total bacterial cell numbers. In addition, the effects of bacterial types and dietary treatments on the correlations of the three methods were also determined.

## 2. Results and Discussion

Correlation coefficients (*n* = 160, 5 diet × 4 replicates × 4 bacteria × 2 PCR reaction) between four methods of expressing the quantification results of digesta bacteria using real-time PCR are shown in Table 1. As mentioned previously, the quantity of bacteria for the control group using the ΔΔCt method and the Pfaffl equation is considered as 1; thus, only the other five dietary treatments were included in the calculations of correlation coefficients.

To further examine any possible effects of bacterial strain or type of diet on the consistency of the four methods, the correlation coefficients were also calculated for different bacterial groups and experimental diets. Correlation coefficients between the four methods in different bacteria populations (*n* = 40, 5 diet, 4 replicates and duplicated PCR reactions) are shown in Table 2.

There were significant (*P* < 0.001) correlations between the four methods for all bacteria groups. The surprising finding was the 1.00 correlation coefficient between the relative method and the Pfaffl equation for all bacterial groups. However, dietary treatments affected the correlation coefficients (Table 3, *n* = 32, 4 bacteria group × 4 replicates × 2 PCR reactions). There was no significant correlation between the Pfaffl equation and the relative and ΔΔCt methods in chickens fed a control diet plus MOS (Bio-Mos,® Alltech, Nicholasville, Kentucky USA) as a mannan oligosaccharides rich prebiotic. There was also no significant correlation between the ΔΔCt or Pfaffl methods and the two other methods in chickens fed a diet containing low shell PKE.

The correlation coefficients between the bacterial groups expressed based on the different methods (*n* = 40, 5 diet, 4 replicates and duplicated PCR reactions) are shown in Table 4. The only inconsistency between the three methods is the correlation between Lactobacilli and Enterobacteriaceae families.

The correlation for the absolute method was positive and highly significant (*P* < 0.001) compared to the lower positive correlation (*P* < 0.05) in the relative and Pfaffl methods. However, there was no correlation between these two bacteria groups in the ΔΔCt method.

The ΔΔCt method was originally developed by Livak and Schmittgen [29] to calculate relative gene expression using real-time PCR. In this method, it is assumed that the expression of a reference gene (housekeeping gene like beta-actin) is independent of external factors and that its expression is quite constant. In this study, as reported by Feng *et al.* [28], we used the Ct of total bacteria in a similar role as the reference genes, and the result showed a lower compatibility with the common absolute method for the expression of bacterial quantity (for example, as cell number per mg digesta). Further examination of the insignificant correlations between the ΔΔCt or Pfaffl method and the other two methods in the dietary group of low shell PKE revealed that the treatment group with the lowest total bacteria quantity and the highest total bacterial population (cell number per mL of digesta liquid phase) was observed in the Control + MOS treatment. The total bacteria in other treatments as a percentage of the Control + MOS treatment were 75% for normal PKE, 80% for enzyme-treated PKE, 89% for enzyme-treated low shell PKE and only 56% for the low shell PKE. Therefore, the assumption of a constant level for total bacteria content in a sample, which is a default value in the calculation of ΔΔCt method, is not appropriate.

The quantity of target bacteria in the ΔΔCt method was calculated as:

$$2^{-((\text{Ct of target bacteria in treated group}-\text{Ct of total bacteria in treated group})-(\text{Ct of target bacteria in control}\;\text{group}-\text{Ct of total bacteria in control}\;\text{group})}$$

A simple calculation using the above formula for the ΔΔCt method suggested that for each unit increase in the Ct value of the total bacteria in a digesta sample, the quantity of target bacteria will increase by two fold.

The Pfaffl equation was designed as a simple method to calculate gene expression without requiring an internal dilution curve. The equation used in this method takes into account the effects of the efficiency of amplification. Because of the high variation in the amplicon size for the different bacterial groups in this study, the use of the Pfaffl method seems logical. In each bacterial group, there was a complete correlation between the Pfaffl equation and the relative method (Table 2). Although the calculations were different, both methods are based on the ratio between a special bacterial group and the total bacterial content of the same digesta sample:

$$\begin{array}{c} \text{Relative bacterial quantity}=\frac{\text{Absolute quantity}\;\text{of the target bacterial population in the}\;\text{digesta sample}}{\text{Absolute quantity of}\;\text{the total bacterial population in the digesta}\;\text{sample}}\\ \text{Pfaffl equation}=\frac{{\left(\text{Efficiency of amplification}\;\text{for target bacterial group}\right)}^{\left(Ct\text{B of birds fed control diet}-Ct\text{B of birds fed experimental diet}\right)}}{{\left(\text{Efficiency of amplification for total bacterial population}\right)}^{\left(Ct\text{TB}\;\text{of birds fed control diet}-Ct\text{TB of}\;\text{birds fed experimental diet}\right)}} \end{array}$$

where, *Ct*B = Threshold cycle for the target bacterial group and *Ct*TB = Threshold cycle for total bacterial population.

However, when the correlation coefficient between the two methods was calculated for all of the bacterial groups (Table 1), the correlation coefficient was less but still significant (0.68). This variation could have resulted from the differences in the amplification efficiencies for the different bacterial groups.

As noted earlier, the lowest total bacterial population was observed in the digesta samples of chickens fed enzyme-treated low shell PKE. Surprisingly, in these birds, the calculating methods resulted in two distinct data groups. There were good compatibilities within the two ΔCt-based methods (ΔΔCt and Pfaffl methods) and within the two non-ΔCt based methods (absolute and relative methods) but not between the two groups. Both the ΔΔCt and Pfaffl methods use the control group as a base for calculations, and because of the exponential nature of the formula, the final results will be affected by differences in the bacterial populations (target or total bacteria) between the treated and control groups. Therefore, there are no correlations between ΔCt-based and non ΔCt-based methods in birds with the lowest total bacterial population (birds fed enzyme-treated low shell PKE diet).

On the other hand, the Pfaffl method was not compatible with the other methods in the digesta samples with the highest total bacterial content (birds fed the control diet plus MOS). This observation again confirms the weakness of the ΔCt-based methods in quantifying bacterial population using real-time PCR.

## 3. Experimental Section

### 3.1. Chickens and Housing

Newly hatched commercial male broiler chickens (Cobb 500) were obtained and maintained in battery pens at the Poultry Research Unit of the Department of Animal Science, University Putra Malaysia. The chicks were provided with free access to water and feed. Six isocaloric and isonitrogenous experimental diets, including a commercial diet as the Control, Control diet plus MOS as a mannan oligosaccharides rich prebiotic, and diets containing four different types of PKE: normal PKE, enzyme-treated PKE, low shell PKE or enzyme treated low shell PKE. Birds were slaughtered at the age of 42 d, and ileal digesta were collected from a total of 12 birds per treatment. Within treatment, an equal amount of the digesta from three birds were pooled and obtained for a total of four replicate samples per treatment for DNA extraction. Approximately 2 g of the pooled digesta was placed in a 2-mL microcentrifuge tube and stored at −20 °C until DNA extraction.

### 3.2. DNA Extraction

DNA was extracted from digesta samples and pure cultures using the QIAamp DNA Stool Mini Kit (Qiagen Inc., Valencia, CA, USA) according to the manufacturer’s protocols. The extracted DNA was stored at −20 °C for later use. The extracted DNA from pure cultures was used to produce a high concentration of the target DNA using normal PCR and to prepare a standard curve. PCR products were purified using the MEGAquick-spin™ (Intron Biotechnology, Inc.), and the purity and concentration of DNA in each sample was measured using a Nanodrop ND-1000 spectrophotometer. The number of copies of a template DNA per mL of elution buffer was calculated using the formula that is available online [30]. Standard curves were constructed using serial dilution of PCR products from pure cultures of each bacterial group.

### 3.3. Quantitative Real Time PCR

The following primers (10 ng/mL concentration) were used to quantify different bacteria populations: for total bacteria, F-5′-CGGCAACGAGCGCAACCC-3′ and R-5′-CCATTGTAGCACG TGTGTAGCC-3′ [31]; for *Lactobacilli*, F-5′-CATCCAGTGCAAACCTAAGAG-3′ and R-5′-GATC CGCTTGCCTTCGCA-3′ [32]; for E*scherichia coli*, F-5′-GTGTGATATCTACCCGCTTCGC-3′ and R-5′-AGAACGCTTTGTGGTTAATCAGGA-3′ [33]; for E*nterococcus* genus, F-5′-CCCTTATTGTT AGTTGCCATCATT-3′ and R-5′-ACTCGTTGTACTTCCCATTGT-3′ [34]; and for Enterobacteriaceae family, F-5′-CATTGACGTTACCCGCAGAAGAAGC-3′ and R-5′-CTCTACGAGACTCAAGCTTG C-3′ [35]. Real-time PCR was performed with the BioRad CFX96 Touch (BioRad, USA) using optical grade plates.

Five nanograms of digesta DNA was added to a 25 μL PCR reaction in a SYBR green assay (iQTMSYBR Green Supermix, BioRad, USA). Each reaction included 12.5 μL SYBR Green Supermix, 1 μL of each Primer, 1 μL of DNA samples and 9.5 μL H_2_O. The reaction conditions for amplification of DNA were 94 °C for 5 min and then 40 cycles of 94 °C for 20 s, 58 °C (*Lactobacillus*) or 60 °C (other bacteria) for 30 s and 72 °C for 20 s. To confirm the specificity of amplification, melting curve analysis was carried out after the last cycle of each amplification. The expected size of amplified fragments were 566 bp for *Eubacteria*, 341 bp for *Lactobacillus* group, 82 bp for *Escherichia coli*, 144 bp for *Enterococcus* genus and 195 for Enterobacteriaceae family and were verified on a 2% (wt/vol) agarose gel for 40 min at 80 V.

Real-time PCR data for each bacteria quantity were calculated in the following three ways:

1. Relative calculation using the delta delta Ct method, (2^−ΔΔCt^): This method was used to calculate the relative abundance (fold changes) of each bacterial group. The threshold cycle, Ct, is the point at which fluorescence above the background is detectable. ΔCt was calculated as the difference between the Ct value with the primers to a specific group of bacteria and the Ct value with the primers to total bacteria. ΔΔCt is defined as the difference between the ΔCt value of each treatment and the ΔCt value of control group. The values derived from the 2^−ΔΔCt^ method show the fold changes of bacterial abundance in a treated sample relative to those of the control sample. The 2^−ΔΔCt^ value of control samples is equal to 1.
2. Pfaffl method: the following equation was developed by M.W. Pfaffl [36] for simple gene expression calculation:
  $$\text{ratio}=\frac{{E_{\text{target}}}^{\Delta \text{Ct target }(\text{control }-\text{treated})}}{{E_{\text{ref}}}^{\Delta \text{Ct ref }(\text{control }-\text{treated})}}$$

For bacterial quantification, the formula is *E*_target_ = Efficiency of amplification of the amplicon of the target bacteria; ΔCt _target_ = the diference between Ct of target bacteria in the treated group and the control group; *E*_ref_ = Efficiency of amplification of the amplicon of the total bacteria; ΔCt _ref_ = the diference between Ct of target bacteria in the treated group and the control group

#### Absolute quantification

**Figure 1:**
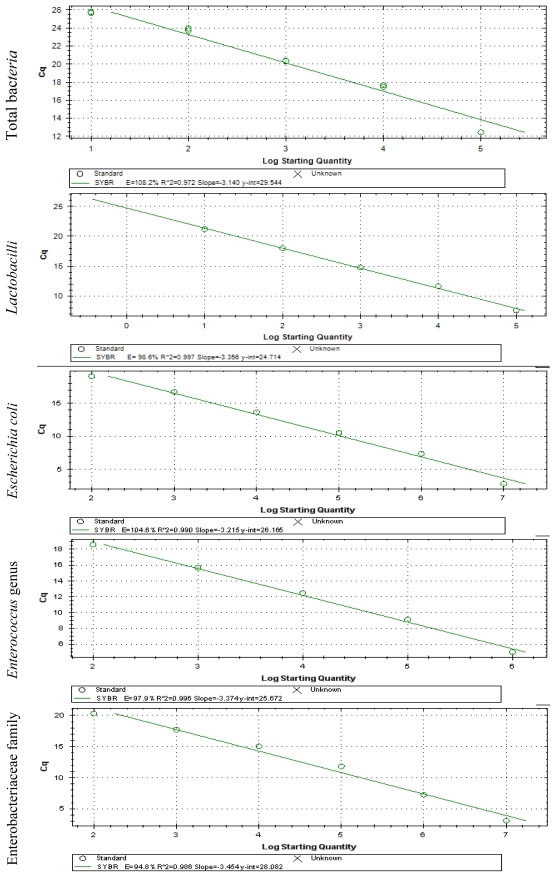
Application curve for serial 10-fold diluted DNA curve of different bacterial groups.

**Figure 2:**
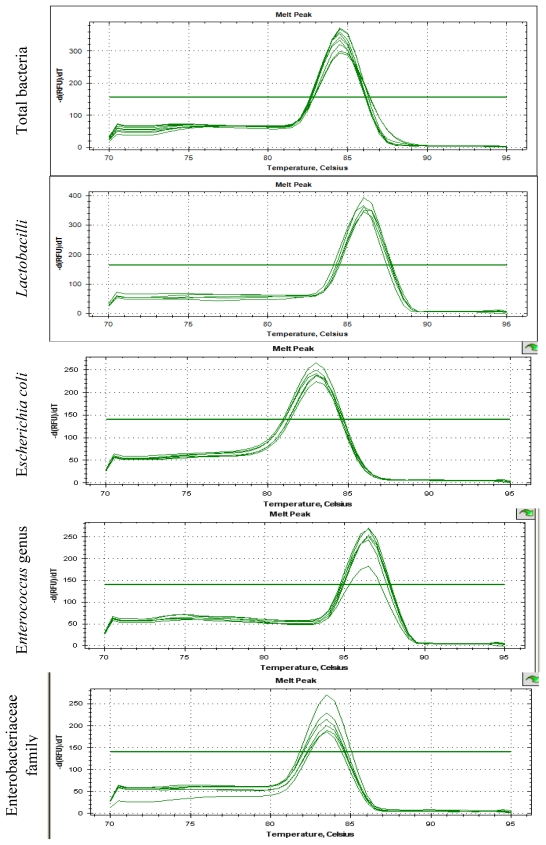
Melting curves for serial 10-fold diluted DNA of different bacterial groups.

Plasmid DNA of *Methanobrevibacter ruminantium*, obtained from cloning process using TOPO TA cloning Kit (Invitrogen), was used to prepare the standard for total bacteria. To prepare the standards for the other groups of bacteria, DNA extracts from pure cultures of *Lactobacillus brevis*, *Escherichia coli*, *Enterococcus faecium* and *Enterobacter cloacae* were used. To calculate the amount of DNA in digesta samples, calibration standards constructed by amplifying known amounts of target DNA were first used to convert the Ct values into quantities of DNA. The *Ct* values for the calibration standards were regressed onto the log10 DNA, allowing a different equation for each run. The functions describing the relationship between *Ct* (threshold cycle) and *x* (log copy number) for the different assays were *Ct* = −0.327*x* + 15.191; *R*^2^ = 0.97 for total bacteria; *Ct* = −0.266*x* + 9.5329; *R*^2^ = 0.99 for *Lactobacilli; Ct* = −0.2991*x* + 10.98; *R*^2^ = 0.99 for *Escherichia Coli; Ct* = −0.2904*x* + 10.354; *R*^2^ = 0.99 for *Enterococcus* genus and *Ct* = −0.2761*x* + 10.784; *R*^2^ = 0.98 for Enterobacteriaceae. The estimated values were expressed as bacteria cell number per mL of liquid phase of digesta. The standard curves presented a dynamic range of 5–6 orders of magnitude and a strong linear correlation (*R*^2^ values were all above 0.98) (Figure 1). The melting curves in Figure 2 show the specificity of amplification for different bacterial groups.

#### Relative quantification

Calculated as a simple ratio between absolute quantities of each bacteria and total *Eubacteria*.

### 3.4. Statistical Analyses

Statistical analyses were performed with the statistical analysis software (SAS version 9.1; SAS Institute Inc., Cary, NC, USA) [37]. Pearson correlation coefficients were used to measure the linear relationship between the results of the three methods for quantitating real-time PCR results.

## 4. Conclusions

The results of this study suggest that using the ΔΔCt or Pfaffl method to express real-time PCR quantitative data for bacteria populations is not compatible with the common absolute method or the simple relative method when dietary treatments affect the total bacteria content of digesta samples. Because of the effect of the total bacteria population on the results obtained from the ΔCt-based methods, it seems that these methods lack the acceptable consistency to be used as valid and reliable methods in real-time PCR-based bacterial quantification studies. Therefore, because of the variable composition of digesta samples, a simple ratio of the cell number of specific bacteria to the corresponding total bacteria cells of the same sample can be a more accurate method to quantify a sample’s population.

## Figures and Tables

**Table 1:** Correlation coefficients between four methods used to express the quantity of bacteria using real time PCR.

	Absolute	Relative	ΔΔCt	Pfaffl equation
**Absolute**	1	0.90353 ***	0.50829 ***	0.58
**Relative**		1	0.5541 ***	0.68
**ΔΔCt**			1	0.83
**Pfaffl equation**				1

*** *P* ≤ 0.001.

**Table 2:** Correlation coefficients between three methods in different bacteria population.

	Absolute	Relative	ΔΔCt	Pfaffl equation
***Lactobacilli***				
Absolute	1	0.89234 ***	0.73279 ***	0.92 ***
Relative		1	0.74956 ***	1.00 ***
ΔΔCt			1	0.75 ***
Pfaffl equation				1
***Escherichia Coli***				
Absolute	1	0.9671 ***	0.8779 ***	0.97 ***
Relative		1	0.89296 ***	1.00 ***
ΔΔCt			1	0.88 ***
Pfaffl equation				1
***Enterococcus*genus**				
Absolute	1	0.75135 ***	0.786604 ***	0.79 ***
Relative		1	0.813478 ***	1.0 ***
Delta			1	0.83 ***
Pfaffl equation				1
**Enterobacteriaceae**				
Absolute	1	0.91871 ***	0.81444 ***	0.92 ***
Relative		1	0.88137 ***	1.00 ***
ΔΔCt			1	0.88 ***
Pfaffl equation				1

*** *P* ≤ 0.001.

**Table 3:** Correlation coefficients between three methods in different dietary treatments.

	Absolute	Relative	ΔΔCt	Pfaffl equation
**Control diet plus Biomos**				
Absolute	1	0.94776 ***	0.60564 ***	NS
Relative		1	0.57425 ***	NS
ΔΔCt			1	NS
Pfaffl equation				1
**Enzyme treated PKE**				
Absolute	1	0.87717 ***	0.43018 *	0.66 ***
Relative		1	0.49445 ***	0.73 ***
ΔΔCt			1	0.82 ***
Pfaffl equation				1
**Low shell PKE**				
Absolute	1	0.82177 ***	NS	NS
Relative		1	NS	NS
ΔΔCt			1	0.85 ***
Pfaffl equation				1
**Enzyme treated low shell PKE**				
Absolute	1	0.96891 ***	0.69688 ***	0.86 ***
Relative		1	0.70467 ***	0.90 ***
ΔΔCt			1	0.78 ***
Pfaffl equation				1
**Normal PKE**				
Absolute	1	0.74926 ***	0.59179 ***	0.56 ***
Relative		1	0.72884 ***	0.89 ***
ΔΔCt			1	0.71 ***
Pfaffl equation				1

NS = non significant;

* *P* ≤ 0.05;

** *P* ≤ 0.01;

*** *P* ≤ 0.001.

**Table 4:** Correlation coefficients between bacteria groups expressed based on different methods.

	Lactobacilli	Escherichia Coli	Enterococcus genus	Enterobacteriaceae family
**Absolute**				
*Lactobacilli*	1	NS	0.50069 **	NS
*Escherichia Coli*		1	NS	0.8389 **
*Enterococcus* genus			1	NS
Enterobacteriaceae family				1
**Relative**				
*Lactobacilli*	1	NS	0.37394 *	NS
*Escherichia Coli*		1	NS	0.91656 **
*Enterococcus* genus			1	NS
Enterobacteriaceae family				1
**ΔΔCt**				
*Lactobacilli*	1	NS	NS	NS
*Escherichia Coli*		1	NS	0.94177 **
*Enterococcus* genus			1	NS
Enterobacteriaceae family				1
**Pfaffl equation**				
*Lactobacilli*	1	NS	0.37 *	NS
*Escherichia Coli*		1	NS	0.91 ***
*Enterococcus* genus			1	NS
Enterobacteriaceae family				1

NS = non significant;

* *P* ≤ 0.05;

** *P* ≤ 0.01;

*** *P* ≤ 0.001.
